# Effectiveness of biologics for reducing occlusive mucus plugs in patients with severe asthma: a systematic review

**DOI:** 10.1186/s12931-026-03501-z

**Published:** 2026-01-21

**Authors:** Helena Aegerter, Christopher E Brightling, Eleanor M Dunican, Bart N Lambrecht, Njira L Lugogo, John D Newell, Celeste Porsbjerg, Sarah Svenningsen, Deborah Clarke, Andrew W Lindsley, Lars Nordenmark, Christopher S Ambrose, Mario Castro

**Affiliations:** 1 Center for Inflammation Research, Laboratory of Immunoregulation and Mucosal Immunology, VIB-UGent Center for Inflammation Research, Ghent, Belgium; 2 Department of Internal Medicine and Pediatrics, Faculty of Medicine and Health Sciences, Ghent University, Ghent, Belgium; 3 Institute for Lung Health, National Institute for Health and Care Research, Leicester Biomedical Research Centre, University of Leicester, Leicester, UK; 4 School of Medicine, University College Dublin, Dublin, Ireland; 5 Department of Medicine, Division of Pulmonary and Critical Care Medicine, University of Michigan, Ann Arbor, MI USA; 6 Department of Radiology, University of Iowa, Iowa City, IA USA; 7 Department of Respiratory Medicine and Infectious Diseases, Bispebjerg Hospital, Copenhagen University, Copenhagen, Denmark; 8 Division of Respirology, Department of Medicine, McMaster University, Hamilton, ON Canada; 9 Firestone Institute for Respiratory Health, St Joseph’s Healthcare, Hamilton, ON Canada; 10 Respiratory and Immunology, BioPharmaceuticals Medical, AstraZeneca, Cambridge, UK; 11 US Medical Affairs, Amgen, Thousand Oaks, CA USA; 12 Late-stage Development, Respiratory and Immunology, BioPharmaceuticals R&D, AstraZeneca, Oslo Norway; 13 Respiratory and Immunology, BioPharmaceuticals Medical, AstraZeneca, Gaithersburg, MD USA; 14 Division of Pulmonary, Critical Care and Sleep Medicine, School of Medicine, University of Kansas, Kansas City, KS USA

**Keywords:** Mucus plugs, Severe asthma, Biologics, Tezepelumab, Dupilumab, Mepolizumab, Benralizumab, Omalizumab

## Abstract

Asthma is a heterogeneous disease characterized by chronic airway inflammation and reversible airflow obstruction. Particularly in severe asthma, airway mucus plugs can contribute to substantial and persistent airflow obstruction, despite inhaled corticosteroid and bronchodilator treatment. Consequently, it is important that clinicians assess and treat mucus plugs. Increased mucus production and airway eosinophilia caused by type 2 (T2) inflammation contributes to mucus plug formation and persistence. Several biologics are available to target T2 inflammation in asthma and studies have described their effects on airway mucus plugs using mucus plug scoring derived from computed tomography scans. However, the outcomes, designs and populations of the various studies have not been comprehensively summarized. A literature search was performed to identify primary publications examining the effects of biologics on mucus plugs in patients with moderate-to-severe asthma, organizing studies by design and study population. Three placebo-controlled randomized controlled trials (RCTs) were identified; one RCT of tezepelumab in patients across baseline blood eosinophil counts (BECs) and fractional exhaled nitric oxide (FeNO) levels and two RCTs of dupilumab in those with elevated BECs or sputum eosinophils and/or elevated FeNO levels. Across these RCTs, biologic treatment decreased mucus plug scores compared with placebo. In the tezepelumab RCT, greater effects were observed in patients with T2-high asthma, highlighting the association between mucus plugging and T2 inflammation. Among T2-high populations, effects were of a similar magnitude across biologics studied. Other biologics (benralizumab, mepolizumab, omalizumab and reslizumab) were evaluated in observational studies without a placebo control, demonstrating reductions in mucus plug scores after treatment. In several studies, decreases in mucus plugs with biologic treatment were associated with improvements in functional outcomes, including pre-bronchodilator forced expiratory volume in 1 second (pre-BD FEV_1_), air trapping, ventilation defects assessed by magnetic resonance imaging, asthma control and health-related quality of life. All studies showed residual plugs after biologic intervention, demonstrating a need for further understanding of how best to quantify and characterize mucus plugs to predict their response to treatment and develop optimal, individualised treatment strategies. This review highlights the relevance of assessing and targeting mucus plugs in clinical practice to help optimise patient outcomes.

## Introduction

Asthma is a heterogeneous disease characterized by chronic airway inflammation, with symptoms of wheezing, shortness of breath and chest tightness caused by variable expiratory airflow obstruction [1]. Symptoms and airflow limitation vary over time and in intensity and are often exacerbated by environmental triggers including viruses, dust mites, moulds, pollens, smoke and other airborne particles [1]. Most patients with asthma have mild-to-moderate disease that can be managed with a treatment combination of inhaled corticosteroids (ICS) and long-acting β2 agonists (LABAs) [1, 2]. However, 3–10% of patients with asthma have severe disease that is uncontrolled despite optimized therapy with high-dose ICS/LABA or that worsens when high-dose inhaled treatment is decreased [1, 2].

In patients with severe asthma, the presence of airway mucus plugs can contribute to persistent airflow obstruction [3, 4]. These mucus plugs comprise elastic, sticky mucus that cannot be expectorated, physically obstructing the airways and contributing to ongoing inflammation through localized airway hypoxia, accumulation of inflammatory mediators and release of pro-inflammatory cytokines [3, 4]. A high number of mucus plugs in patients with severe asthma correlated with decreased lung function and irreversible airway obstruction [5, 6]. In studies of patients enrolled in the Severe Asthma Research Program (SARP), 68% of patients with severe asthma had occlusive airway mucus plugs [5], and 82% of patients with mucus plugs at baseline had detectable mucus plugs 3 years later [7]. For many patients with mucus plugs, airflow obstruction persisted despite systemic corticosteroid and bronchodilator (BD) treatment [5].

Mucins are polymeric glycoproteins that make up the principal solids in mucus. MUC5B and MUC5AC are the major gel-forming mucins in airways and are largely responsible for the viscoelastic biophysical properties of mucus [8]. MUC5B is the predominant mucin found in the healthy airway, secreted from submucosal glands and secretory cells, while MUC5AC produced by goblet cells is released into the airway lumen following allergen exposure, infections, or autonomic stimulation [8, 9]. The cysteine-rich domains in MUC5AC are susceptible to disulfide bridges, forming a tightly cross-linked mesh network that is simultaneously tethered to the epithelium, resulting in tenacious mucus [10, 11]. Mucus plugs can arise through a combination of goblet cell metaplasia-driven mucus hypersecretion, altered mucus composition and impaired mucus clearance [8]. In addition, eosinophil peroxidase (EPO) and thyroid peroxidase (TPO) from epithelial cells can catalyse oxidation of thiocyanate by hydrogen peroxide (H_2_O_2_) to produce hypothiocyanite, which drives the cross-linking of mucins and stiffening of mucus gel through the oxidation of mucin cysteine residues [5, 9]. Furthermore, the eosinophil product galectin-10 (also known as Charcot–Leyden crystal protein, CLC-P) can spontaneously auto-crystallize in the mucus-rich environment to form Charcot–Leyden crystals (CLCs) in the airway lumen after eosinophil activation and cell death, inducing mucus secretion, inflammation and immunoglobulin E (IgE) production [12].

**Fig. 1:**
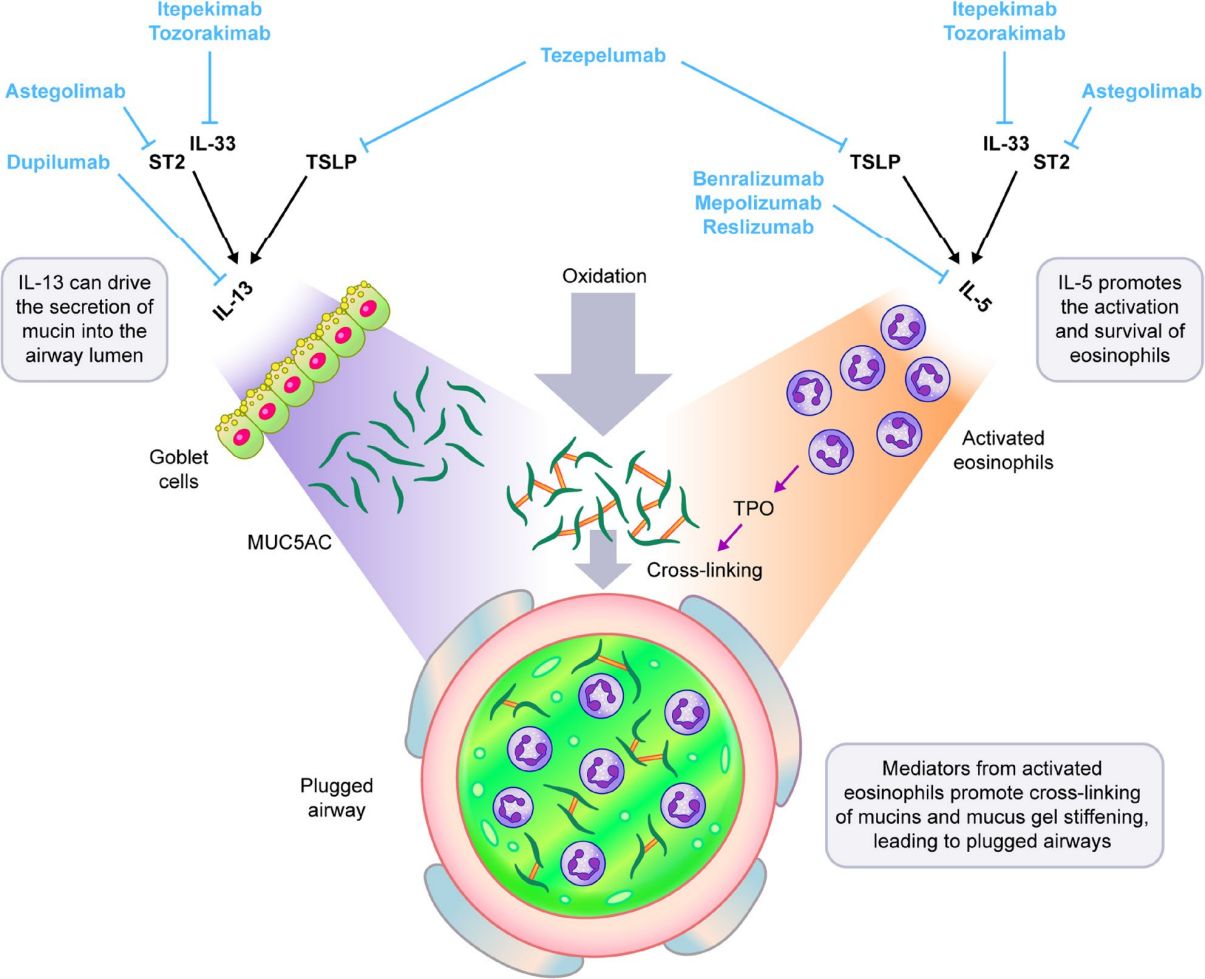
The role of T2 inflammation in the formation of mucus plugs, with potential target sites of biologics. IL, interleukin; MUC5AC, mucin 5AC; ST2, serum stimulation-2; T2, type 2; TPO, thyroid peroxidase; TSLP, thymic stromal lymphopoietin. Adapted from Dunican EM et al. Ann Am Thorac Soc. 2018;15(Suppl 3):S184–91 [13]

Although our understanding of mucus plug biology is not complete, several immune factors have been identified that correlate with mucus plugs, including sputum eosinophilia and high levels of interleukin (IL)-5, IL-13 and thymic stromal lymphopoietin (TSLP), which are features of type 2 (T2) inflammation (Fig. 1) [3, 5, 6, 13, 14]. Goblet cell metaplasia and subsequent mucin hypersecretion are typically mediated by T2 cytokines, including IL-13, which can contribute to the increase in MUC5AC production seen in patients with T2-high asthma [8, 15]. IL-13 can also induce TPO, increase H_2_O_2_ production and induce the expression of pendrin, an exchanger that transports thiocyanate into the airway lumen, further promoting mucin cross-linking [5, 9, 13]. IL-13 also decreases the ciliary beat frequency of ciliated human epithelial cells, and promotes tethering of MUC5AC-rich mucus gels to the epithelium in vitro, both of which may contribute to the impaired clearance of mucus in the airways [10, 16, 17]. In addition, IL-13 reduces the number and function of ciliated cells in cultures of human epithelial cells, which may explain the decrease in ciliated cell number observed in patients with severe asthma compared with healthy individuals [17]. IL-13 induces interlectin-1 gene expression, which is highly expressed in patients with T2-high asthma. Interlectin-1 gene and protein expression are decreased by a common genetic variant that is associated with protection from the formation of mucus plugs in T2-high asthma [18]. High levels of IL-5 promote the survival and accumulation of eosinophils in the airway lumen, which also contributes to mucus stiffening [13]. TSLP can drive all of these pathways given its role in increasing IL-5 and IL-13 activity in the airways [19]. Non-T2 pathways may also contribute to mucus plugs in asthma and other obstructive airway diseases. For example, neutrophilic inflammation can drive the formation of neutrophil extracellular traps, which have been observed in the airways of patients with asthma and can increase human mucus viscoelasticity in vitro [20]. Pathological examination of plugs also often reveals the presence of cross-linked fibrin, suggesting that coagulation pathways are also triggered [21].

Advances in the methodologies used to assess mucus plugs and their functional consequence have enabled more robust evaluation of the potential effects of treatments on mucus plugs. Computed tomography (CT) scans allow for non-invasive imaging of the lungs. A mucus plug is defined as a complete occlusion of the airway lumen, with the density of the plug comparable to adjacent pulmonary vessels and a clearly patent (air containing) airway lumen both proximal and distal to the mucus plug. In CT scans, mucus plugs appear as areas of opacification within the airway lumen, contiguous with the patent airway lumen across sequential transverse CT slices [5]. To ensure optimal image quality while maintaining the lowest possible radiation exposure, a standardized, multi-detector, high-resolution CT protocol is recommended. Images should be acquired with 110–120 kVp and reconstructed using a neutral kernel (for example B35, B, STANDARD and FC01/FC17 for Siemens, Philips, GE and Toshiba scanners, respectively). A high spatial resolution is required, with a suggested isotropic voxel size of 0.5 mm and a maximum recommended slice thickness of 1.5 mm. An image quality control process is needed to confirm protocol adherence and to exclude scans with motion or metal artifacts, or submaximal inspiration.

A visual scoring system has been developed for quantifying mucus plugs using CT in clinical research [5]. Lung segments may be systematically examined, with a score of 1 assigned to each segment containing one or more mucus plugs. The segment scores can then be summed to generate a total mucus score. Early studies defined 20 segments − 10 in the right lung and 10 in the left lung [5]. However, many recent trials have adopted the 18-segment model as described by the Netter atlas [19, 22] with 10 lung segments in the right lung and 8 segments in the left lung. The 18-segment model aligns more closely with the segmental airway anatomy, making it more robust and well defined in the CT images. A limitation of these approaches is that within a single segment, one or numerous plugs are counted the same, regardless of their size or the airway generation(s) they obstruct; as a result, mucus plug burden may not be completely captured. To ensure best practice, two independent readers should review each CT scan, with a third reader providing an independent assessment of each lung segment where the first and second reader disagree on the presence of a mucus plug. In large trials, a full overread of all scans may not be feasible and a strategy for a double read of a subset of scans may be required. In routine clinical care, there is unlikely to be more than one reader, removing the safety net that is in place in clinical trials to ensure best practice. CT scans may be paired with a regional assessment of airflow obstruction using hyperpolarized ^129^Xe ventilation magnetic resonance imaging (MRI) scans to assess the functional consequence of mucus plugs [6, 23, 24]. The burden of ventilation defects observed by ^129^Xe ventilation MRI is quantified as the ventilation defect percent (VDP), which is correlated with the CT mucus score and presence of mucus plugs at the level of the bronchopulmonary segment [6, 24].

Several biologics that target T2 inflammation in asthma are available or are in development. Monoclonal antibodies that target IgE or T2 cytokines and their receptors include astegolimab (anti-serum stimulation-2 [ST2/IL-1RL1/IL-33R]), benralizumab (anti-IL-5 receptor), dupilumab (anti-IL4/IL-13 receptor), itepekimab (anti-IL-33), lunsekimig (anti-TSLP/IL-13), mepolizumab (anti-IL-5), omalizumab (anti-IgE), reslizumab (anti-IL-5), tezepelumab (anti-TSLP) and tozorakimab (anti-IL-33) [25–28]. Although several biologic therapies that target T2 inflammation have been shown to be beneficial for patients with severe, uncontrolled asthma, the specific effects of biologics on mucus plugs have been less well reported [19, 29]. Interpretations of the effects of biologics on mucus plugs are complicated by differences in study designs and study populations. The aim of this literature review was to comprehensively and objectively examine the effects of biologics on mucus plugs in patients with moderate-to-severe asthma, organizing studies by design and study population.

## Methods

### Literature search

Ovid (MEDLINE and Embase databases) was searched on 2 December 2024 to identify primary publications (abstracts and manuscripts) and review articles that examined the effects of biologics (astegolimab, benralizumab, dupilumab, itepekimab, mepolizumab, omalizumab, reslizumab, tezepelumab and tozorakimab) on mucus plugs in patients with severe asthma. The search string was: (“severe asthma” and [“mucus plug” or “mucus plugging”) and (“biologic” or “astegolimab” or “benralizumab” or “dupilumab” or “itepekimab” or “mepolizumab” or “omalizumab” or “reslizumab” or “tezepelumab” or “tozorakimab”]). There were no publication date or language restrictions. Duplicate search results were excluded from the analysis. Case reports, editorials and encores of abstracts already identified in the literature search were excluded from the analysis. Furthermore, if an abstract was superseded by a primary manuscript that was also identified in the literature search, the abstract was excluded from the analysis. This review is based on previously conducted studies and does not contain any new studies with human patients or animals performed by any of the authors.

## Results

### Literature search

The literature search returned 54 publications, which were reviewed for inclusion suitability. Thirty-seven of these publications did not meet the inclusion requirements. Of the 17 articles included in the final analysis, nine were primary publications (eight manuscripts, one abstract) [19, 29–36] and eight were review articles [8, 25, 37–42]. The review articles were assessed to identify any studies investigating the effect of biologics on mucus plugs in severe asthma that may have been missed in the literature search. However, no additional studies were identified. Therefore, the findings are based on the nine primary publications identified in the literature search. All primary publications included in the analysis used CT lung scans to assess mucus plugs [19, 29–35]. Three publications examined paired CT scans with assessment of ventilation using hyperpolarized ^129^Xe MRI scans [30, 34, 35].

### Summary of the effect of biologic treatment on mucus plugs

**Fig. 2:**
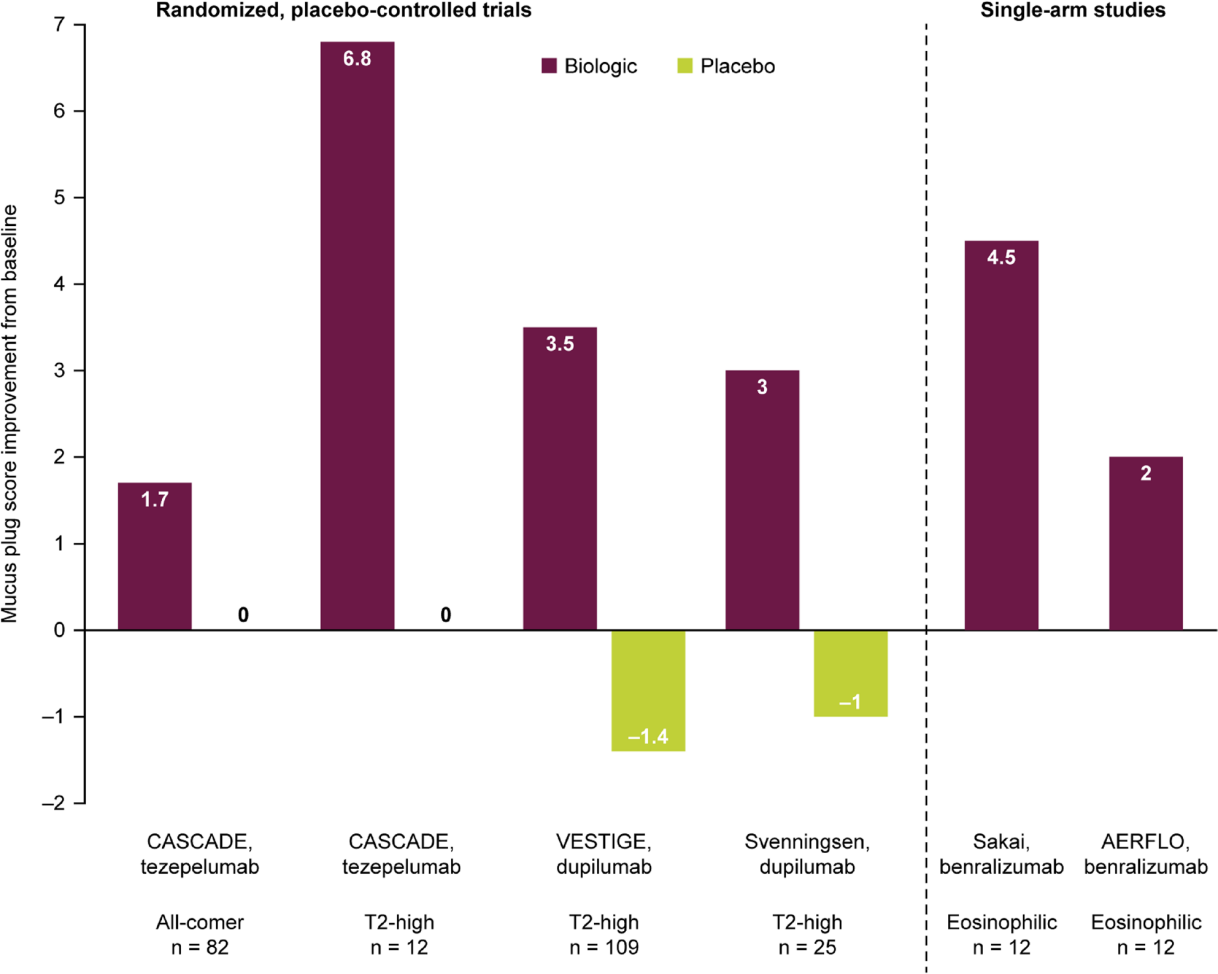
Change in mucus plug score from baseline with biologic treatment from randomized, placebo-controlled trials and single-arm studies. Error bars are not shown as the units for error data differed across studies; error data are provided in Tables 1 and 2, where available Mucus plug score ranges from 0–18 for CASCADE, VESTIGE and Sakai; 0–20 for Svenningsen and AERFLO. PRISM was not included owing to the difference in scale T2, type 2

Of the nine primary publications included in the analysis, three publications reported data from placebo-controlled randomized controlled trials (RCTs) of biologics (Table 1; Fig. 2). Of these RCT publications, one reported data for tezepelumab and two RCTs investigated dupilumab. One RCT identified in the literature search reported data on mucus plugs from a population of patients with moderate-to-severe, uncontrolled asthma that was not enriched based on inflammatory biomarker levels. This study, CASCADE (NCT03688074), was an exploratory, mechanistic, placebo-controlled study of tezepelumab, with no restriction on participant blood eosinophil counts (BECs) or fractional exhaled nitric oxide (FeNO) level at screening [19]. CASCADE used a capping approach to include approximately 30% of participants with BECs of ≤ 150 cells/µL, approximately 30% with BECs of 150 to ≤ 300 cells/µL, and approximately 40% with BECs of ≥ 300 cells/µL at screening [19]. In contrast, the two RCTs of dupilumab had inclusion criteria based on the enrichment of inflammatory biomarker levels at screening. VESTIGE (NCT04400318) was a placebo-controlled, randomized, double-blind study of dupilumab in patients with moderate-to-severe, uncontrolled asthma with elevated T2 biomarkers (baseline BECs of ≥ 300 cells/µL and FeNO ≥ 25 ppb) [29]. Dupilumab was also assessed among patients with moderate-to-severe, uncontrolled asthma and T2 inflammation (baseline FeNO > 25 ppb or BECs of ≥ 300 cells/µL or ≥ 3% sputum eosinophils) in a single-centre, randomized, double-blind, placebo-controlled study (NCT03884842) [30]. It is, however, important to note that the three studies differed in the starting number of mucus plugs or the baseline mucus plug score, with a higher mean mucus plug score in the single-centre study compared with both the CASCADE and VESTIGE studies.

**Table 1:** Randomized controlled trials identified by the literature search that investigate the effect of biologics on mucus plugs

Biologic	Trial/analysis	Design	Study size	Patient characteristics	Endpoint assessed	Technique used to assess mucus plugs	Average mucus plug score at baseline	Change from baseline in mucus plug score
Tezepelumab 210 mg SC Q4W	CASCADE (NCT03688074) [19, 43]	Double-blind, randomized, placebo-controlled study	*N* = 82; tezepelumab *n* = 37; placebo *n* = 45	Patients (aged 18–75 years) with uncontrolled, moderate-to-severe asthma	Change in mucus plug score from baseline to EOT	CT	Median (min, max): tezepelumab, 1.0 (0, 12); placebo, 0.0 (0, 9)	Absolute change (mean ± SD): tezepelumab, − 1.7 ± 2.6; placebo, 0.0 ± 1.4
			Tezepelumab, *n* = 5; placebo, *n* = 7	Those above with characteristics of T2 inflammation^a^			Mean (min, max): 3.46 (0, 12)	Absolute change (mean):^b^ tezepelumab, − 6.8; placebo, 0.0
Dupilumab 300 mg Q2W	VESTIGE (NCT04400318) [29]	Double-blind, randomized, placebo-controlled study	*N* = 109 patients; dupilumab, *n* = 72; placebo, *n* = 37	Patients (aged 18–70 years) with uncontrolled moderate-to-severe asthma and elevated T2 biomarkers^a^ and with pre-BD ppFEV1 ≤ 80%, with ≥ 1 severe asthma exacerbation in the year before screening	Mucus airway plugging, mucus airway volume, airway inflammation, and related lung function changes at week 24	CT	Mean (SD): dupilumab, 7.2 (5.1); placebo, 6.9 (5.0)	Absolute change: dupilumab, − 3.5; placebo, 1.4 LS mean difference (95% CI): −4.9 (− 6.5, − 3.3)
Dupilumab 600 mg loading dose followed by 300 mg SC Q2W	Svenningsen et al. (NCT03884842) [30]	Single-centre, double-blind, randomized, placebo-controlled study	*N* = 25; dupilumab, *n* = 13; placebo, *n* = 11^c^	Uncontrolled moderate-to-severe asthma and T2 inflammation^d^	Prospectively defined exploratory chest CT and ^129^Xe ventilation MRI outcomes at week 16	CT and ^129^Xe MRI	Data not reported	Absolute change (95% CI): dupilumab, − 3 (–6 to − 1); placebo, 1 (–1 to 2) Between-treatment difference (95% CI): –4 (–7 to − 1); *p* = 0.018

^a^Baseline BEC of ≥ 300 cells/µL and FeNO of ≥ 25 ppb

^b^These data are unpublished; however, because the data were available to the authors, they were included to enable comparisons with published data from other studies

^c^Of 25 patients, 13 patients who received dupilumab and 11 patients who received placebo were included in the efficacy analysis

^d^Baseline FeNO of > 25 ppb or BECs of ≥ 300 cells/µL or ≥ 3% sputum eosinophils

Mucus plug score ranges 0–18 for CASCADE and VESTIGE; 0–20 for Svenningsen

*BD* bronchodilator, *BEC* blood eosinophil count, *CI* confidence interval, *CT* computed tomography, *EOT* end of treatment, *FeNO* fractional exhaled nitric oxide, *FEV1* forced expiratory volume in 1 s, *LS* least-squares, *MRI* magnetic resonance imaging, *pp* percent predicted, *Q2* Wevery 2 weeks, *Q4* Wevery 4 weeks, *SC* subcutaneous, *SD* standard deviation, *T2* type 2

Among patients with CT scans at baseline and end of treatment (EOT) in CASCADE (*n* = 82), the mean (standard deviation [SD]) absolute change from baseline in mucus plug scores (range: 0–18) was − 1.7 (2.6) in patients who received tezepelumab and 0.0 (1.4) in patients who received placebo. At EOT, 38% of patients in the overall tezepelumab group compared with 13% in the overall placebo group transitioned from having at least one mucus plug to having no mucus plugs [19]. Among those without plugs at baseline, 3% of tezepelumab recipients versus 16% of placebo recipients had mucus plugs at EOT [19]. The greatest reduction in mucus plug score was observed in patients with dual characteristics of T2 inflammation (baseline BEC of ≥ 300 cells/µL and FeNO ≥ 25 ppb), with a mean change from baseline in mucus plug score of − 6.8 in patients who received tezepelumab (*n* = 5) compared with 0.0 in patients who received placebo (*n* = 7) [19, 43].

**Fig. 3:**
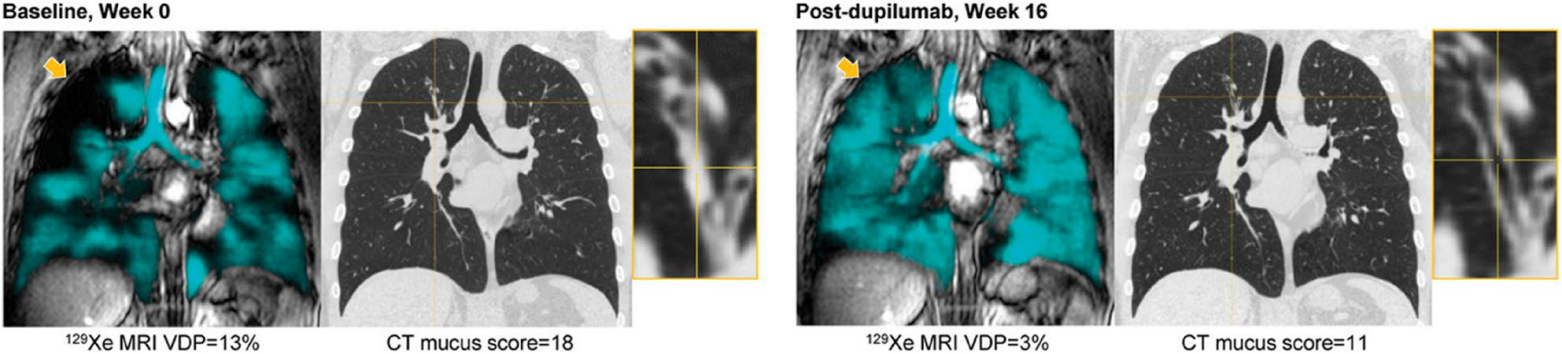
^129^Xe ventilation MRI (cyan) and CT coronal slices at baseline and the end of dupilumab treatment (week 16) for a 40-year-old man. CT mucus score and MRI ventilation were improved but not normalized by dupilumab. Yellow crosshairs identify a mucus plug in the apical segment of the right upper lobe and a distal ventilation defect (arrow) at baseline that were resolved after dupilumab administration. Adapted with permission from Svenningsen S et al. *Am J Respir Crit Care Med* 2023;208:995–997 [30] CT, computed tomography; MRI, magnetic resonance imaging; VDP, ventilation defect percentage

In VESTIGE (*n* = 109), patients with moderate-to-severe, uncontrolled asthma with multiple elevated T2 biomarkers (baseline BECs of ≥ 300 cells/µL and FeNO ≥ 25 ppb) who were treated with dupilumab had reduced mucus plug scores (range 0–18), with a least-squares mean difference (95% confidence interval [CI]) from baseline of − 4.9 (− 6.5, − 3.3) points compared with placebo (nominal *p* < 0.001) at week 24 [29]. Importantly, a CT scan performed at week 4 also demonstrated a reduction in mucus plug score with dupilumab compared with placebo. Furthermore, air trapping was numerically reduced in those treated with dupilumab while increases were seen in those who received placebo at week 24 [29]. A similar effect on mucus plugs was also observed in the single-centre RCT of dupilumab, which investigated patients with moderate-to-severe, uncontrolled asthma and T2 inflammation (baseline FeNO > 25 ppb or BECs of ≥ 300 cells/µL or ≥ 3% sputum eosinophils). At week 16, patients treated with dupilumab (*n* = 13) had reduced mean mucus plug scores (range 0–20) compared with placebo (*n* = 10), with a between-treatment difference of − 4 (95% CI: −7, − 1; *p* = 0.018) [30]. Figure 3 (reproduced from Svenningsen S, *et al.)* presents an example of improvement in ventilation and mucus plugging visualized by MRI and CT coronal slices after dupilumab administration [30].

The other six primary publications included in the analysis were single-arm studies without placebo controls (Table 2). In the first, CT scans were assessed for mucus plugs in 27 patients with severe asthma after the initiation of dupilumab. Mucus plugs were significantly decreased at 4 months, although specific mucus plug scores were not reported [31]. Mucus plugs were also decreased from baseline in 28 patients with moderate-to-severe asthma treated with dupilumab at week 48 (*p* = 0.02) in a prospective, observational study, although the change in mucus score was not provided [36]. Patients with severe asthma were also investigated in the PRISM study, a prospective, observational, multicentre study in South Korea. Patients with severe asthma and characteristics of T2 inflammation were eligible for a range of biologics (benralizumab, dupilumab, mepolizumab, omalizumab and reslizumab). Thirty-four patients treated with biologics had CT scans at baseline and follow-up (10–12 months), with a decrease in the mucus plug score (based on 0, 1, 2 or 3 points for absence or involvement of 1–5, 6–9 or > 9 segments, respectively) observed at follow-up by two independent readers (median [interquartile range], reader 1, − 2.0 [− 1.0, 0.0], *p* < 0.001; reader 2, − 0.5 [− 1.0, 0.0], *p* = 0.001) [32].

**Table 2:** Single-arm studies identified by the literature search that investigate the effect of biologics on mucus plugs

Biologic	Trial/analysis	Design	Study size	Patient characteristics	Endpoint assessed	Technique used to assess mucus plugs	Effect of treatment on mucus plugs
Dupilumab	Koya et al. Study location: Niigata University Medical and Dental Hospital [31]	Single-arm	*N* = 27	Severe asthma, defined as any of the following: > 2 exacerbations per year, Asthma Control Test score < 20, or predicted values of FEV_1_ < 80% despite use of high-dose inhaled steroids plus controllers	Effect of dupilumab on mucus plugs	CT	Mucus plugs decreased significantly after the introduction of dupilumab. Specific scores were not reported
Dupilumab	Tajiri T et al. Study location: Nagoya City University [36]	Prospective, single-arm observational study	*N* = 30, *n* = 28 with severe asthma	Adult patients with moderate-to-severe asthma (GINA guidelines) [1]	Change from baseline to week 48 in CT mucus plug scores	CT	Mucus plug score decreased from baseline to week 48 (*p* = 0.02). Specific scores were not reported
Biologic treatment (mepolizumab, reslizumab, benralizumab, dupilumab, or omalizumab)	Precision Medicine Intervention in Severe Asthma study (PRISM) [32]	Prospective, observational, multicentre study (10–12-month follow-up)	*N* = 182; *n* = 34 with initial and follow-up CT scans	Korean patients aged 18–80 years with severe asthma (ERS/ATS 2014 definition [2]). Patients with a T2 inflammation phenotype were eligible for biologic treatment	Not stated	CT	The median (IQR) change in mucus plugging extent was − 2.0 (− 1.0, 0.0), *p* < 0.001 for reader 1 and − 0.5 (− 1.0, 0.0), *p* = 0.001 for reader 2
Benralizumab	Sakai et al. Study location: Niigata University Medical and Dental Hospital [33]	Single-arm	*N* = 12	Severe, uncontrolled, eosinophilic asthma, (ERS/ATS 2014 definition [2])	The number of mucus plugs before and after 4–6 months of benralizumab	CT	The number of mucus plugs significantly decreased 4 months after the introduction of benralizumab (median mucus plug count: pre-treatment, 5.5; post-treatment, 1; *p* = 0.001)
Benralizumab	AERFLO (NCT03733535) [34, 35]	Single-arm	*N* = 29 with uncontrolled asthma; *n* = 27 with mucus plug data at baseline: *n* = 12 with mucus plug data at 2.5 years	Patients (aged 18–80 years) with poorly controlled asthma (GINA guidelines) [1] exacerbation history within 4 weeks of enrolment. Inclusion criteria included a baseline BEC of ≥ 300 cells/µL, and patients with no response to oral corticosteroids but with a baseline BEC of < 300 cells/µL could be enrolled if they had clinically significant sputum (> 2%) or blood eosinophilia (> 150 cells/µL) in the past 12 months	Change in ^129^Xe MRI ventilation defect percentage	CT and ^129^Xe MRI	Mean (SD) mucus plug score at baseline: 3 (4) Mean (SD) mucus plug score at 2.5 years: 1 (1), *p* = 0.03

Mucus plug score ranges from 0–18 for Sakai; 0–20 for AERFLO; 0–3 for PRISM (based on absence or involvement of 1–5, 6–9 or over 9 segments, respectively)

*ATS* American Thoracic Society, *BEC* blood eosinophil count, *CT* computed tomography, *ERS* European Respiratory Society, *FEV*_*1*_forced expiratory volume in 1 s, *GINA* Global Initiative for Asthma, *IQR* interquartile range, *MRI* magnetic resonance imaging, *SC* subcutaneous, *SD* standard deviation, *T2* type 2

In another single-arm study, 12 patients with severe, uncontrolled, eosinophilic asthma (European Respiratory Society [ERS]/American Thoracic Society [ATS] 2014 definition [2]) had a significant decrease in median mucus plug count (score range: 0–18) 4 months after the initiation of benralizumab (pre-treatment, 5.5; post-treatment, 1; *p* = 0.001) [33]. AERFLO (NCT03733535) was an open-label, single-arm, pilot study in patients with severe, poorly-controlled eosinophilic asthma (BECs of ≥ 300 cells/µL) that investigated the effect of benralizumab on airway mucus and ventilation, assessed by ^129^Xe MRI [34, 35]. Mucus plugs were significantly decreased at 2.5 years of follow-up, with a mean (SD) mucus plug score (range: 0–20) of 3 (4) at baseline (*n* = 27) and 1 (1) at 2.5 years (*n* = 12, *p* = 0.03) [35].

### Summary of the associations between mucus plugs and markers of T2 inflammation after biologic treatment

Several of the primary publications identified in the literature search reported associations between changes in mucus plugs after treatment with biologics and markers of T2 inflammation. In the CASCADE study of patients with moderate-to-severe, uncontrolled asthma, reductions in mucus plugs in tezepelumab recipients were correlated with reductions in BEC (Spearman’s rank correlation coefficient [*ρ*]: tezepelumab, 0.28; placebo, 0.09) and in plasma and bronchoalveolar lavage eosinophil-derived neurotoxin, a biomarker for eosinophilic degradation (*ρ*: tezepelumab, 0.51 and 0.47, respectively; placebo, − 0.05 and − 0.30, respectively) [19].

In another study, a correlation was reported between the change in mucus plug score and serum IgE levels in patients with severe asthma treated with dupilumab [31]. Reductions in mucus plug scores were also correlated with reductions in FeNO levels and sputum eosinophil proportions from baseline to week 48 in patients with moderate-to-severe asthma treated with dupilumab [36]. Mucus plug scores were positively associated with sputum eosinophil levels at baseline in the PRISM study of patients with severe asthma and T2 inflammation (reader 1, *ρ* = 0.566; reader 2, *ρ* = 0.551; all *p* = 0.001); however, a correlation between change in sputum eosinophil levels and change in mucus plug scores at follow-up was not assessed [32]. In patients with severe, uncontrolled eosinophilic asthma treated with benralizumab, there was not a significant correlation at follow-up between changes in mucus plug counts and changes in BEC (*ρ* = 0.48, *p* = 0.107) or sputum eosinophil percentage (*ρ* = 0.39, *p* = 0.155). However, this study included a small sample size of 12 patients and, therefore, had limited statistical power [33].

### Summary of the associations between mucus plugs and functional outcomes after biologic treatment

Several of the primary publications reported associations between changes in mucus plugs after treatment with biologics and functional outcomes. Reductions in mucus plug scores were correlated with increases in pre-BD forced expiratory volume in 1 s (FEV_1_) in patients with moderate-to-severe asthma treated with tezepelumab (*ρ*, tezepelumab, − 0.51; placebo, 0.02) [19]. Patients with mucus plugs receiving tezepelumab demonstrated a significant improvement in percent predicted pre-BD FEV_1_ compared with placebo (12.7% vs. 4.4%), whereas patients without mucus plugs did not (2.2% vs. 6.0%). This suggests that tezepelumab may improve lung function, at least in part, through the reduction of mucus plugs. Similarly, improvements in pre-BD FEV_1_ from baseline to follow-up were associated with decreases in mucus plug scores in patients with moderate-to-severe asthma and T2 inflammation who were treated with dupilumab in the VESTIGE study (Pearson’s correlation coefficient − 0.62; *p* < 0.001) [29]. Improvements in pre-BD FEV_1_ were also associated with a decrease in mucus plug score, as assessed by two independent radiologists, in patients with severe asthma and T2 inflammation treated with biologics in the PRISM study (*ρ*, reader 1, − 0.51; reader 2, − 0.52; *p* < 0.05) [32]. A positive correlation was also observed between the percentage reduction in mucus plug counts and the change in percent FEV_1_ after treatment in patients with severe eosinophilic asthma receiving benralizumab (*ρ* = 0.68, *p* = 0.015) [33].

In patients with moderate-to-severe asthma and T2 inflammation treated with dupilumab, a decrease in mucus plugs was associated with improved airflow assessed by ^129^Xe ventilation MRI, with the change in the ^129^Xe MRI VDP significantly correlated with the change in mucus plug score (*ρ* = 0.61; *p* = 0.027) [30]. In patients with severe, poorly controlled eosinophilic asthma (BECs of ≥ 300 cells/µL) who were treated with benralizumab, there was a significant improvement at day 28 in the VDP in participants with five or more mucus plugs at baseline (*n* = 9; *p* = 0.006) [34]. Given that mucus plug scores at day 28 were not assessed in this study, a causal relationship between mucus plugs and ventilation defects could not be confirmed [34]. A follow-up analysis at 2.5 years of benralizumab treatment showed a significant improvement in VDP (*p* = 0.003) and mucus plug scores (*p* = 0.03); however, a correlation between these two parameters was not assessed [35].

A reduction in mucus plugs after dupilumab treatment was reported to correlate with changes in Asthma Quality of Life Questionnaire score in patients with severe asthma [31] and in patients with moderate-to-severe asthma [36]. In VESTIGE, dupilumab versus placebo treatment showed a significant improvement in mucus plug score (*p* < 0.001) and Asthma Control Questionnaire (ACQ)-7 score (*p* < 0.001); however, a correlation between these two outcomes was not assessed [29]. A positive correlation was observed between the percentage reduction in mucus plug counts and changes in Asthma Control Test scores after treatment in patients with severe eosinophilic asthma receiving benralizumab (*ρ* = 0.74; *p* = 0.006) [33]. Similarly, in a multivariate analysis, mucus plug score at day 0 was a significant variable for change in ACQ-6 score at 28 days (*p* = 0.001) and 2.5 years (*p* = 0.009) of benralizumab treatment in patients with poorly controlled eosinophilic asthma [34, 35].

## Discussion

This review demonstrates that biologic treatments that block T2 cytokines and mediators can reduce airway mucus plugs in some patients with severe asthma. The highest-quality evidence comes from three randomized, placebo-controlled trials involving either tezepelumab or dupilumab. Greater treatment effects were observed in patients with T2-high asthma than in the overall population, and among the T2-high population, treatment effects were of similar magnitude across biologics. Other biologics (benralizumab, mepolizumab, omalizumab and reslizumab) were only evaluated in observational studies without a placebo control, which demonstrated reductions in mucus plug scores of varying magnitude after treatment. However, these results must be interpreted with caution given that mucus plugs can be stable in the airways for several years [5, 44] and improvements in mucus plug scores can occur owing to natural fluctuations in disease. Longitudinal studies are needed to assess the timeframe of mucus plug resolution. As a result, it is unknown to what degree improvements observed after treatment initiation in single-arm studies can be causally attributed to treatment. Evidence of a direct effect on mucus plugs is weakest for mepolizumab, omalizumab and reslizumab, because these biologics were not evaluated in isolation in any identified study.

Several studies reported associations between decreases in mucus plugs and improvements in functional outcomes of clinical relevance. Overall, these results validate the role of T2 inflammation in mucus plug formation and persistence and support the relevance of the assessment of mucus plugs in clinical practice. In addition, the fact that some patients did not resolve mucus plugs during biologic treatment, and even demonstrated more plugs at the end of biologic treatment, raises the questions of whether some mucus plugs are resistant to current biologics due to incomplete T2 blockade and whether non-T2 mechanisms contribute to mucus plug formation and persistence. Individual results should be captured and displayed to correctly report this heterogeneity of response, for example, by using line graphs of pre- and post-treatment mucus plug scores. Longitudinal studies are also needed to assess the timeframe of mucus plug resolution.

Although this review focuses on asthma, mucus plugs are also observed in other airway diseases, including chronic obstructive pulmonary disease (COPD), non-cystic fibrosis bronchiectasis (NCFB), cystic fibrosis and fungal airway diseases [45–47]. Mucus plugs are frequent in COPD and have been identified in more than half of smokers in a study of patients with this condition [48]. High mucus plugs correlate with higher exacerbation risk, reduced symptom control, decreased pulmonary function and all-cause mortality in COPD, compared with lower mucus plugs [8, 48]. Sputum from patients with NCFB has been shown to be hyper-concentrated with mucin, suggesting a dysregulation of mucin hydration that may contribute to disease progression [49]. However, it is not known to what extent the biological mechanisms that contribute to mucus plugs overlap between asthma, COPD and NCFB, and whether biologics targeting T2 inflammation would also decrease mucus plugs in other airway diseases. In the FRONTIER-4 study (NCT04631016) of COPD patients with chronic bronchitis on dual- or triple-inhaled maintenance therapy, there was a trend of reduced mucus plug score in patients receiving tozorakimab compared with placebo (least-squares mean difference: − 1.5; 80% CI: − 3.0, 0.0; *p* = 0.097) [50]. Therapeutics that target non-T2 pathways of mucus plug formation should also be investigated further. For example, macrolides, such as azithromycin, are broad-spectrum antibacterials that can inhibit airway epithelium mucus secretion in vitro and in vivo and have reduced COPD exacerbations in clinical trials [51, 52]. Future research is needed to determine the applicability of these results to other airway diseases.

Advances in mucus plug quantification and characterization methods will facilitate the assessment of mucus plugs in large-scale clinical trials. For example, mucus plugs can have ‘stringy’ or ‘stubby’ phenotypes, determined by their length, with high mucus plug burden more often attributed to the ‘stringy’ phenotype [44]. In addition, several automated mucus plug scoring algorithms are in development, but these require further validation [53, 54]. Comprehensive mucus plug annotation on CT provides additional quantitative information, such as the total number of mucus plugs, mucus volume or mass and obstructed airway cross-sectional area. Comprehensive mucus plug scoring potentially has greater sensitivity for the assessment of treatment effects and the functional impact on airway physiology.

## Conclusions

This literature review describes the effects of biologics on mucus plugs in patients with asthma as reported in studies of varying design and study populations. All the primary publications identified in this literature search used CT scans to assess mucus plugs, with a subset of studies correlating mucus plug data to ventilation assessed by ^129^Xe MRI. Three placebo-controlled RCTs of patients with moderate-to-severe asthma were identified; one RCT did not exclude patients based on baseline BECs or FeNO, whereas two RCTs only enrolled those with elevated BECs or elevated sputum eosinophils and/or elevated FeNO levels. Treatment decreased mucus plug scores in all three RCTs. Five single-arm studies, with varying study populations, also reported a decrease in mucus plugs after biologic treatment. Decreases in mucus plugs after biologic treatment correlated with decreases in T2 inflammation markers and air trapping, and increases in lung function, ventilation, asthma control and health-related quality of life. These findings support the clinical relevance of mucus plugs in moderate-to-severe asthma. Future research should explore the applicability of these results to other airway diseases, including COPD and NCFB, and assess if mucus burden is a predictor of biologic response. Additionally, data on the extent of mucus plug resolution required to have clinically meaningful improvements in functional outcomes would be valuable in clinical practice.

## Data Availability

Data sharing is not applicable to this article as no datasets were generated or analysed during the current study.

## Abbreviations

BD: bronchodilator
BEC: blood eosinophil count
CLC: Charcot–Leyden crystals
COPD: chronic obstructive pulmonary disease
CI: confidence interval
CT: computed tomography
EPO: eosinophil peroxidase
EOT: end of treatment
FeNO: fractional exhaled nitric oxide
FEV_1_: forced expiratory volume in 1 s
ICS: inhaled corticosteroid
IgE: immunoglobulin E
IL: interleukin
LABA: long-acting β2 agonist
LS: least-squares
MRI: magnetic resonance imaging
NCFB: non-cystic fibrosis bronchiectasis
Q2W: every 2 weeks
Q4W: every 4 weeks
RCT: randomized controlled trial
SARP: Severe Asthma Research Program
SC: subcutaneous
SD: standard deviation
T2: type 2
TPO: thyroid peroxidase
TSLP: thymic stromal lymphopoietin
VDP: ventilation defect percent
H2O2: hydrogen peroxide
ST2: serum stimulation-2
